# Cases of Gout, with Observations

**Published:** 1818-03

**Authors:** William Balfour

**Affiliations:** Edinburgh


### *Cases of Gout, with Observations.* By WILLIAM BALFOUR, M. D. Edinburgh.

(Continued from vol. iv. p. 171.)

CASE VI. About the beginning of July last, Mrs. G. was attacked with a sense of coldness, numbness, and obtuse pain in her left foot and ankle, and at times, though slightly, in the leg. In this state she continued a month, without using any other remedy than flannel and the warm bath; from which she derived no benefit. About the beginning of August, the pain in the sole of the foot, heel, and ankle became extremely acute, attended with swelling, redness, and constitutional derangement. She now put herself under the care of an experienced practitioner, who prescribed rubefacients, blisters, two full bleedings from the arm, saline purgatives, calomel, and other preparations of mercury, which produced ptyalism of some days' continuance,—and these in the order in which I have enumerated them. In the course of a month, nearly three weeks of which she was confined to bed, the inflammatory symptoms were subdued; and, in the horizontal posture, she was free from pain. But, whenever she put her foot to the ground, and attempted to bear upon it, she found her cure was merely palliative. She could not walk but with excruciating pain. Being now informed by her Doctor, that her complaint was a combination of rheumatism and gout, for the complete cure of which he could not limit a period; and being unable to perform the functions of her office, she gave up her situation (that of a housekeeper), and came to Edinburgh in great dejection of mind. A lady, nearly connected with the family she had left, strongly advised her to put herself under my care, which she did on the 1st of September.

I found my patient not altogether free from fever; but her chief complaint was severe pain, on attempting to walk, in the sole of the foot, extending from near the first joint of the great toe along the under and inner side of its metatarsal bone, round the heel-bone, outer margin of the malleolus externus, and under the tendo Achillis its whole length. The moment I touched her foot I perceived a compressible tumour on the inner and under side of the metatarsal bone of the great toe. This circumstance was not known to the patient before, and she was much chagrined at it, as foreboding incurable lameness. On the whole, I found, that, in this instance, I had "tender ground" to tread upon indeed.

As my patient appeared to be an intelligent, sensible woman, I thought it proper to inform her, not only of the mode of treatment I meant to pursue, but of the opinion of some "observant pathologists," that such treatment must occasion the translation of the disease from the extremities to vital organs. Upon stating my reasons, however, for being of a contrary opinion, I was invited to proceed in my own way. I therefore applied compression and percussion in my usual manner, but found the affections submitted to my care obstinate as they were acute. Nearly a week elapsed before the tumour was dissipated, and the pain arising from it was subdued. Not but that the effects of every operation evinced the efficiency of the means, for the patient could bear their force to be increased at every successive application. But if the pain in the sole of the foot was obstinate, that in the heel proved much more so; nor could any impression be made upon it but by mere force of percussion. After having operated for some time, one day, in this manner, I observed to the patient, that her foot was covered with profuse perspiration. She replied, that there was seldom any sensible perspiration in her feet,—a circumstance she considered as very prejudicial to her health,—that she perceived an unusual moisture on her feet during the operation some days before; from which she dated, in her own mind, the melioration of her complaints. To this circumstance, therefore, I afterwards paid particular attention, and found I could promote perspiration in the foot at pleasure, without injuring the parts affected. In two weeks my patient was, in every respect, much better; in three weeks she was perfectly well.

I beg leave to call the attention of my readers, and especially of Dr. Johnson, to the fact of compression and percussion increasing the perspiration of parts to which they are applied, as evinced in this patient's case:—a fact which demonstrates the salutary tendency of my mode of treating rheumatism and gout, and which must have great weight with every candid mind, in regard to the question at issue betwixt Dr. Johnson and me. I have elsewhere said, that a scald is repellent, so compression and percussion are conciliatory. This I have now established on a foundation which all the efforts of my opponents can never shake. A scald is not likely to increase, during its application, the perspiration of any part; neither are compression and percussion, which promote perspiration powerfully, likely to prove in any degree repellent.

Whether gout is a disease of the nervous system, as Drs. Boerhaave and Cullen think; whether it consists in a morbid action of the vessels, induced by a faulty action of the nerves, as Mr. Abernethy (in his Hunterian Lecture, if I recollect rightly) defines disease in general to be; or, that it consists in inflammation and its consequences, affecting peculiarity of structure,—certain it is, that compression and percussion induce a salutary change in the action of both the vessels and nerves of parts affected with gout. Nor is this one whit more inconceivable than many other sufficiently well authenticated facts. Dr. Simpson, of St. Andrew's, in his Dissertations de Re Medica, observed near a hundred years ago, that by the insertion of a foreign body into a wound, this might be converted into a sort of gland, and made to discharge pus for any length of time. We know, that the slightest variation in the circumstances of a wound, whether from a chemical or mechanical cause, will change the action of the secreting surfaces. I have demonstrated, that the suppurative can be instantly changed into the adhesive process, by mere apposition of the surfaces of a wound. Mr. Baynton has proved, that the simple application of a bandage to a limb most powerfully accelerates the healing process in old wounds. Since, then, such apparently trifling causes are seen to produce such mighty effects, in *divided* vessels and nerves, is it incredible, that the morbid action of *continuous* vessels and nerves should be changed into the salutary, by means which cannot fail to affect them so powerfully as do compression and percussion? Mechanical causes often produce chemical effects in the living body. The abstraction of blood, in certain circumstances, affects the qualities of the whole remaining mass. The application of compression prevents the formation of matter in whitlow. The mechanical displacement of fæcal matter from the intestines moderates the action of the whole system.

But I am under no necessity of having recourse to analogy to prove the change of action induced by compression snd percussion in parts affected with gout. The patient is *conscious* of, and the practitioner *sees* the effects of this change of action. Now, as every effect must have a cause, if, by a peculiar mode of handling parts affected with rheumatism and gout, I remove pain, swelling, immobility, must not these effects be the result of a healthy action induced in the parts? This being admitted (and it cannot be denied), it follows, that my practice in rheumatism and gout rests on as sound and rational principles as any improvement that ever was made in the healing art.

As Dr. Johnson has declined the controversy, I shall also now take leave of it; trusting that, on more mature consideration of the subject, that gentleman's candour will triumph over first impressions.

I crave indulgence, however, for a few words in regard to the success of the practice under consideration, in the hands of others; and on this point my hopes are by no means sanguine. This does not arise from the inefficacy of the practice. It is eminently efficacious; and whoever will spend the time and take the pains with their patients that I do with mine, will have all the success that I have stated myself to have had. But if practitioners satisfy themselves with directing their patients or attendants to apply compression and percussion, they will have little or no success, and they have no right to expect it. Whoever does not spend from ten minutes to an hour with his patient, according to the circumstances of the case, has no experience in the matter, and therefore cannot decide either for or against my practice. Practitioners of this description will, most likely indeed, reject it as totally useless, without reflecting that the fault lies with themselves. But such conduct will not alter the nature of things. The conscientious and pains-taking practitioner, who conceives himself bound to do his utmost for the man that confides his life and health to his care, will not be swayed by the opinion of the superficial observer, who gives evidence, he does not wish 'any improvement should be received, unless it proceeds from himself. Of such pitiful conduct I have met with repeated instances. I shall relate one or two as a specimen.—A young lady had a rheumatic affection in her right wrist for eighteen months, all which time she was under the care of an eminent surgeon, who satisfied himself with *ordering* the application of a bandage. The affection grew daily worse, till the patient could neither sew, nor play on the piano-forte. In these circumstances she applied to me, in great fear and dread of offending the family-surgeon. In five days I gave her the complete command of her hand; and, had my plan been adopted at the beginning, this lady's hand would not have been pained a day.

A woman attended an eminent surgeon in this city, for nine months, occasionally, with a rheumatic affection of her right elbow-joint, wrist, and interosseous ligament of the fore-arm. This gentleman *directed* a bandage to be applied to the parts; as if a bandage alone could materially affect synovial surfaces, cartilaginous and ligamentous structures! It is needless to say, the woman was as well at the end of the nine months as she was at the beginning, and not one whit better. By one operation, however, I gave her such command of her hand and arm as astonished her own family and all her neighbours.—Now, as these two gentlemen, to my certain knowledge, pronounce my practice in rheumatism and gout to be "*nonsense*,"* I must

---

* Such was not the behaviour of Mr. John Bell, certainly one of the be permitted to tell *them*, and all *such*, that they are totally ignorant of the matter. They never made trial of the practice. They do not know what compression and percussion mean, as applied to the diseases in question. They are, therefore, not more competent judges of their effects, than a blind man is of colours. Were the case otherwise, how does it happen that I do more for a patient in ten minutes than these gentlemen do in nine months, or in eighteen months, or than, without adopting my practice, they *ever* could do? Whoever believes, that unequal distribution of the fluids and nervous energy is the source of all diseases, or rather constitutes disease; and that the difference of diseases depends on modifications of this inequality as influenced by its cause; will have no difficulty in conceiving, that mechanical powers may be brought in aid of other remedies, in many cases; and that, in other cases, they will constitute the principal remedy. If, as often happens in rheumatism and gout, congestions are formed, which the powers of the constitution, however aided by general remedies, are unable to resolve,—is it not the most natural thing in the world to have recourse to means which directly unload the vessels of the parts in fault? And is not the application of the same means, in the acute form of these diseases, most likely to prove a powerful auxiliary in resolving and preventing congestion, and thereby, of moderating the action of the system?

These observations are for the consideration of those who decry a practice, into the principles of which they never gave themselves the trouble to inquire.

WILLIAM BALFOUR.

*Edinburgh, Nov.* 12, 1817.

---

greatest ornaments of his profession that this or any age has produced. Possessing a superiority of mind which is incompatible with the meanness of professional jealousy, and a generosity of sentiment which disdains all selfish considerations, this gentleman consulted me in the case of a lady, a very near connection of his own, who had been long afflicted with rheumatic and other chronic complaints; and, so far was he from despising my mode of treatment, as some affect to do, who are no more to be compared to him than a glimmering taper to the meridian sun, that he committed the patient to my care in the most polite and obliging manner. Unfortunately, I found the lady in a state of debility that precluded the idea of mechanical operation.

